# Linking strategies and biases when matching cohorts to the National Pupil Database

**DOI:** 10.23889/ijpds.v1i1.369

**Published:** 2017-04-19

**Authors:** Johnny Downs, Efrosini Setakis, Tarek Mostafa, Richard Hayes, Matthew Hotopf, Tamsin Ford, Ruth Gilbert

**Affiliations:** 1 King's College London; 2 University College London Institute of Education; 3 University of Exeter; 4 University College London

## Objectives

To compare sample biases when linking education data to external datasets using opt in and opt out consent models for the Millennium Cohort Study (MCS) - opt in, and the Case Register Interactive Search Child and Adolescent Mental Health sample (CRIS CAMHS) -opt out. 

## Approach

We compared demographic factors in the linked and unlinked populations when two cohorts, the MCS, and CRIS CAMHS data were linked to the National Pupil Database. The MCS is a birth cohort collecting prospective data on the social, economic and health-related circumstances of children surveyed at roughly two-yearly intervals from the age of 9 months. At age 7, parents were asked to consent for information from their child's education records (via the UK Department for Education's National Pupil Database, NPD) to linked to the MCS. Of the 9000 adults with parental responsibility, 8448 (93.9%) consented. The CRIS CAMHS sample is the UK's largest, anonymised clinical database for children and adolescents referred to South London child and adolescent mental health services, which is collected using opt out consent. All 35, 426 children were eligible for linkage to their educational data.

## Results

For MCS, 7446 (82.7% of the population) eligible children were successfully linked to NPD following opt in consent. After stratification into distinct geographical regions, London children in the most deprived quartile of neighbourhood deprivation were over twice as likely (O.R 2.5, C.I 1.18-5.3) not to have their educational records linked. For the opt out CRIS CAMHS cohort, 30,178 (85.1%) were linked, with no significant differences in linkage rates between children in the highest and lowest quartiles of deprivation (O.R 1.05, C.I 0.93-1.18). Relative to children of White ethnicity, Asian, Black African and Mixed ethnic groups were significantly less likely to be matched in both studies. Black Caribbean ethnicity was significantly associated with non-linkage in the MCS cohort (O.R 3.0, C.I 1.49-6.01), but not in CRIS CAMHS (0.96, C.I 0.83-1.12). 

## Conclusion

Record linkages are a valuable enhancement to child-based longitudinal studies and clinical registries, allowing evaluation of questions relevant to public health and social care policy. Opt out consent approaches improve representation of more socially disadvantaged populations. Nevertheless, whether using opt in and opt out consent process, possible biases due to linkage error can be important and need to be addressed when analysing and interpreting results. 

